# Robust Species Distribution Mapping of Crop Mixtures Using Color Images and Convolutional Neural Networks

**DOI:** 10.3390/s21010175

**Published:** 2020-12-29

**Authors:** Søren Kelstrup Skovsen, Morten Stigaard Laursen, Rebekka Kjeldgaard Kristensen, Jim Rasmussen, Mads Dyrmann, Jørgen Eriksen, René Gislum, Rasmus Nyholm Jørgensen, Henrik Karstoft

**Affiliations:** 1 Department of Engineering, Aarhus University, Finlandsgade 22, 8200 Aarhus N, Denmark; msl@eng.au.dk (M.S.L.); rnj@eng.au.dk (R.N.J.); hka@eng.au.dk (H.K.); 2 Department of Agroecology, Aarhus University, Blichers Allé 20, 8830 Tjele, Denmark; rekk@agro.au.dk (R.K.K.); jim.rasmussen@agro.au.dk (J.R.); jorgen.eriksen@agro.au.dk (J.E.); rg@agro.au.dk (R.G.); 3 School of Engineering, Aarhus University, Finlandsgade 22, 8200 Aarhus N, Denmark; madsdyrmann@ase.au.dk

**Keywords:** mixed crop mapping, species composition estimation, targeted fertilization, grass clover mixtures, proximity sensing, precision agriculture, deep learning

## Abstract

Crop mixtures are often beneficial in crop rotations to enhance resource utilization and yield stability. While targeted management, dependent on the local species composition, has the potential to increase the crop value, it comes at a higher expense in terms of field surveys. As fine-grained species distribution mapping of within-field variation is typically unfeasible, the potential of targeted management remains an open research area. In this work, we propose a new method for determining the biomass species composition from high resolution color images using a DeepLabv3+ based convolutional neural network. Data collection has been performed at four separate experimental plot trial sites over three growing seasons. The method is thoroughly evaluated by predicting the biomass composition of different grass clover mixtures using only an image of the canopy. With a relative biomass clover content prediction of R^2^ = 0.91, we present new state-of-the-art results across the largely varying sites. Combining the algorithm with an all terrain vehicle (ATV)-mounted image acquisition system, we demonstrate a feasible method for robust coverage and species distribution mapping of 225 ha of mixed crops at a median capacity of 17 ha per hour at 173 images per hectare.

## 1. Introduction

Crop mixtures of grass and clovers have many advantages such as reduced use of industrial fertilizer, lower production costs, increased protein self-sufficiency, increased yield stability, and improved feed quality [1]. To optimize fertilization of grass clover mixtures, the clover proportion must be known, as the fertilizer response is greater at low clover proportions. Visual inspection of clover proportion is uncertain and impossible when large fields needs to be covered. Therefore, automated and robust methods for estimating clover proportion in mixtures are needed for example to adjust fertilization levels at ‘close to real-time’. Although the technique has been shown to work with deep learning based methods [2,3], there is a need to verify and enhance the stability of model predictions across different growth conditions, camera systems, and mixtures of species and varieties.

The problem of automatically assessing the mixed crop composition using computer vision and top-down images has been investigated during the previous 15 years. Before the introduction of deep learning [4], all approaches were based on morphological operations [5,6,7,8,9] or binary patterns [10]. While the use of morphological operations to distinguish grass from clovers based on the leaf widths proved valuable, it was highly sensitive to factors such leaf sizes, camera setup, lighting, and image resolutions. This led to a need for ongoing parameter-tuning, specific for each acquisition setup and growth stage.

Skovsen et al. [3] introduced the use of deep learning for grass clover segmentation to reduce the need for ongoing model refinements. Using synthetically created training data, an FCN-8s [11] fully convolutional model was trained to segment images into grass, clover, weeds, and soil. Over the duration of three out of four seasonal cuts, the model improved state-of-the-art accuracy in grass clover semantic segmentation, while demonstrating an increased robustness towards changing leaf sizes.

Skovsen et al. [12] proposed a cascaded FCN-8s approach to further segment clovers into species of red clover and white clover. Although the results looked promising for estimating the clover species in the biomass samples, it did not generalize to the first seasonal cut. This was possibly due to a non-uniform vertical distribution of white clover in the high yielding samples.

Bateman et al. [2] proposed an improved model architecture for semantic segmentation, named LC-Net (Local Context Network). Based on a VGG16 model [13] backbone, an encoder–decoder architecture was designed to fuse standard feature maps with contextual feature maps of lower resolution. This resulted in more accurate semantic segmentations, specifically along object boundaries.

Due to the inherent entanglement of the deep learning model architecture, training data, hyper parameters, and image quality, a direct comparison between proposed methods and their results is increasingly inaccurate. As a consequence, an increase or decrease in either of the components greatly influence the final result.

Mapping the spatial species distribution of mixed crops is a difficult task, but necessary, to fully benefit from targeted fertilization. Bakken et al. [14] systematically mapped grass clover plots of sizes up to 4 × 8 m. Ten days after being cut, the plots were fully photographed guided by a metal frame, enclosing each plot. Using morphological operations, the images were then segmented into grasses, clovers, and weeds to determine the species distributions both spatially and temporally. Skovsen et al. [15] presented preliminary large scale species distribution maps of grass clover mixtures. Using an all terrain vehicle (ATV)-mounted camera system, 17,759 images, spatially distributed across 150 ha, were collected and semantically segmented into grass, clover, weeds, and soil pixels using a publicly available FCN-8s model.

In this work, we have three contributions: (1) Extension of biomass-labeled image data for grass clover segmentation evaluation. Compatible with the publicly available GrassClover dataset [16], we extend the number of samples by 110% and double the number of experimental sites; (2) State-of-the-art results in predicting the relative biomass clover content from top-down canopy images in changing conditions. Using the synthetic training data of the GrassClover dataset, an Xception-65 based DeepLabv3+ model was trained to accurately segment real images into grass, clover, weeds, and soil pixels in changing conditions. Then, based on the detected distribution of species in the imaged canopy, the corresponding biomass composition was predicted with higher accuracy than previous methods. (3) Robust large scale species distribution mapping of 225 ha grass clover fields. Based on the improved accuracy and robustness of the proposed method, 29,848 spatially distributed images were semantically segmented to predict the relative clover content at each sample, and then interpolated to maps.

## 2. Material

This study uses images from three sources: (1) images from a high-resolution mirrorless system or digital single-lens reflex (DSLR) camera from plot trials, (2) images from a high-velocity camera mounted on an ATV from large fields, and (3) synthetic images. Each of these three image types has a purpose. The images from the high-resolution system camera were taken in plot trials, and are used to find the correlation between dry weight and image information. The images from the high-velocity camera on the ATV are used to find clover grass variations at field level, and the synthetic images are used to create large amounts of realistic training data. Each of these image types will be covered in the following sections.

### 2.1. Plot Trial Sites

The sample pairs of biomass samples and corresponding canopy images originate from four grass clover plot trials located 60–250 km apart. Each plot trial was designed to answer research questions unrelated to this paper but introduced a large variation with regards to soil conditions, fertilization strategies, seed compositions, and herbicide usage. Due to the variation in growth conditions and composition of crops, we consider that the images are representative of most clover-grass fields. A comparison of the four sites is shown in Table 1. The samples from plot trial sites A and B have previously been used to evaluate grass clover segmentation performance [3,12] and were published as part of a grass clover recognition challenge [16]. The extension of samples from plot trial site C and D is a contribution of this paper and substantially extends the evaluation basis of image based species distribution prediction in terms of sample quantity, weather conditions, locations, and seeded clover and grass species. In total, 915 biomass samples with corresponding canopy image were collected at these four sites.

#### 2.1.1. Biomass Samples in Plot Trials

**Figure 1:**
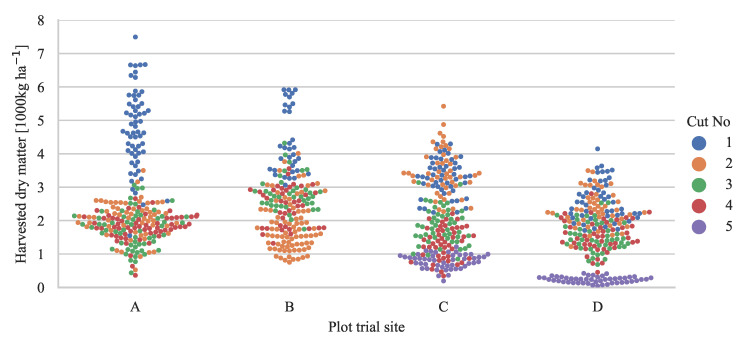
Distribution of biomass samples across quantity, plot trial site, and seasonal cut number. The harvested yield is based on the sampled area of 0.25 m^2^ per biomass sample.

Plant samples were cut with electrical cutters in plots of 0.5 × 0.5 m, in the same plots as the images were taken. The harvested material was hand sorted into four categories: red clover, white clover, grass, and weeds. Each sample category was weighed, dried at 60 °C for 48 h and weighed again to determine dry matter yield and botanical dry matter species composition. The dry matter yield of sample categories with less than 5 g of fresh weight was estimated assuming a 25% dry matter content. The sample weight distribution across plot trial sites and seasonal cuts is shown in Figure 1.

#### 2.1.2. Image Acquisition in Plot Trials

**Figure 2:**
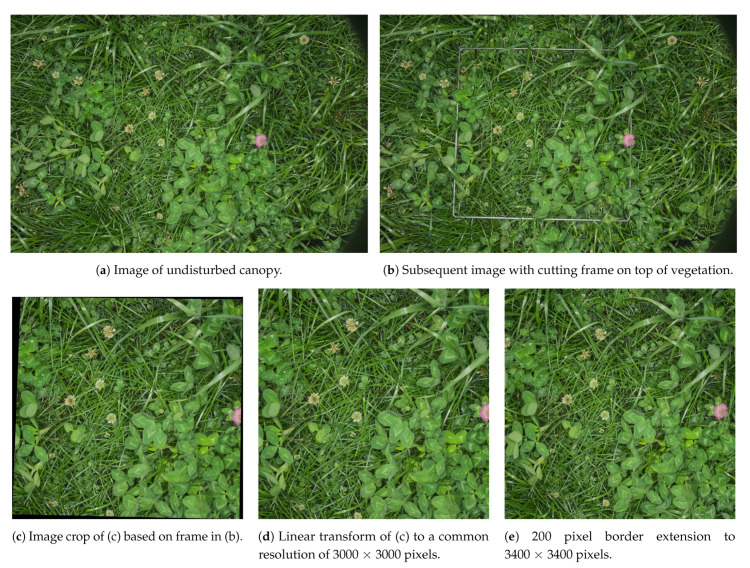
Steps of image preprocessing from captured images to convolutional neural network (CNN)-inference ready images.

Image acquisitions in plot trial C and D were carried out with a Sony (Tokyo, Japan) A7 Mirrorless interchangeable-lens camera (MILC) with a Sony Sonnar T* FE 35 mm F2.8 ZA lens. With the camera mounted on a horizontal rod connecting two tripods, images were captured facing down. Without moving the camera, two images were collected before each biomass sample as shown in Figure 2a,b. The first image captured the undisturbed canopy. The second image included the frame, defining the 0.5 × 0.5 m area to be cut, in the image domain. By placing the frame on top of the vegetation, as opposed to the ground, perspective transformations and height estimations could be omitted from the proceeding processing steps. In addition, 420 biomass samples were photographed using a ring flash to increase the visibility in the images. Due to failure of an external power supply, 60 biomass samples were photographed using two speedlight flashes, mounted on either side of the camera.

#### 2.1.3. Image Preprocessing

All images were manually developed from raw formats, with guidance from a cardboard color calibration target. Image crops, corresponding to each biomass sample of 0.5 × 0.5 m, were then generated as demonstrated in Figure 2. To remove the challenge of scale variance originating from changing canopy to camera distances, each crop was linearly transformed to a square image of a fixed 3000 × 3000 pixels. To reduce edge artifacts in plant classifications from missing context, each crop was extended 200 pixels on all sides, but disregarded after the semantic segmentation.

### 2.2. Large Scale Image Acquisition in Farmed Fields

**Figure 3:**
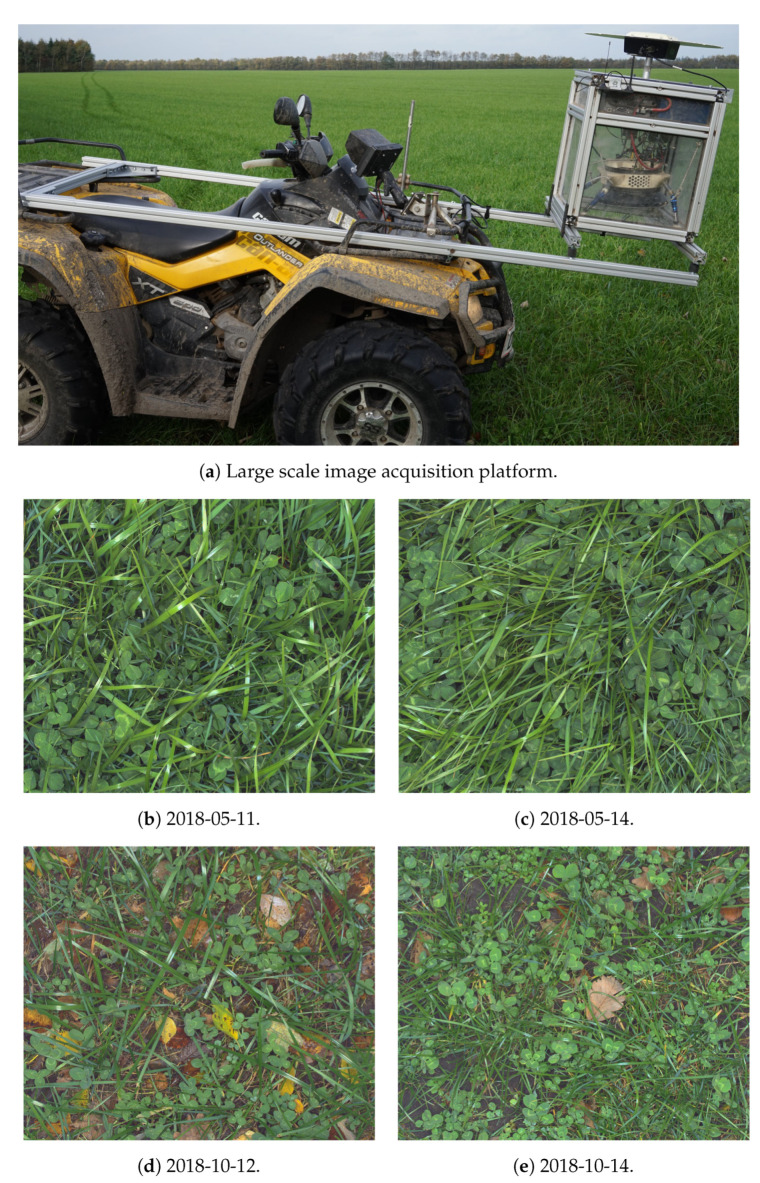
The large scale image acquisition platform and sample images from each day of large scale image acquisition. The difference in yield is noticeable from the larger leaf sizes in the first row, and the visible soil in the bottom row. The images were captured at a velocity of 18 km h^−1^ without motion blur due to the specially developed camera system.

The primary desire of predicting the biomass composition from images is the possibility of estimating the species compositions at scales where manual plant fractioning is unfeasible. To demonstrate the robustness of the proposed method in this context, large scale image acquisitions were carried out at the three dairy farms, located in Jutland, Denmark, with 25–68 km spacing in between. All fields were seeded with perennial ryegrass and white clover following the growers’ conventional procedure. One farmer did not use pesticides for weed control in his fields. Image acquisition was carried out in May, a few days before the high-yielding first cut of the season, and, in October, with sparser vegetation. A summary of the large scale acquisitions is shown in Table 2. The robustness of the system is thus demonstrated in high and low yielding conditions, on both conventionally and organically grown fields with a large difference in the occurrence of weeds. Image samples from each of the four image acquisitions are shown in Figure 3b–e. The image-based grass clover fractioning must therefore be able to handle the diversity between these fields.

#### 2.2.1. ATV-Mounted Image Acquisition Platform

The image acquisition system, shown in Figure 3a, is based on a 5.0 megapixel USB3 Vision camera (Point Grey (Richmond, BC, Canada), GS3-U3-51S5C-C) and a 16 mm lens (Edmund Optics (Barrington, IL, USA), 86-571). Artificial illumination was provided by a ring flash (Alienbees (Paul C. Buff Inc, Nashville, TN, USA), ABR800), the ring flash was modified by adding a light sensor to provide a trigger output for the camera based on the illumination power of the ring flash. This is done to keep exposure time to a minimum, opening the camera shutter just during the very peak of the illumination of the flash. Keeping the exposure time short results in the illumination from the sun to become insignificant, thereby keeping a constant illumination. Furthermore, it also eliminates motion blur for travel velocity less than 70 km h^−1^. The camera is mounted in the center of the ring flash, using a 3D printed adapter. The adapter is made with a series of spring elements to center the camera inside the ring flash. The ring flash itself is mounted on three oil-spring dampeners to protect camera and flash from mechanical shocks. The camera assembly is mounted on the front of the ATV (Can-am, Outlander 500 XT) using aluminum extrusions tied to the front and rear luggage racks. The camera is mounted approximately 0.9 m above the ground. The camera system is controlled by a computer (Nvidia (Santa Clara, CA, USA), TX2) running the Robot Operating System [17]. Images are geotagged based on input from a real-time kinematic global navigation satellite system (RTK-GNSS)(Trimble (Sunnyvale, CA, USA) BD920-W3G receiver with 23903-00 antenna, gpsnet.dk provided the virtual reference station for RTK). Triggering of the flash is based on the location measured by the GNSS, if the distance is larger 5 m from the last image location, a new flash is triggered, which in turn triggers the camera.

#### 2.2.2. Sampling Strategy

Each field was systematically traversed with the ATV-mounted camera at approximately 18 km h^−1^. The image sampling was triggered every 5 m in the driving direction with a typical track-to-track distance of 6–12 m. In October, each field was additionally sampled along the field border prior to the interior sampling.

#### 2.2.3. Image Preprocessing

The images were digitally developed from Bayer pattern images using the approach of Malvar et al. [18]. To minimize the effects of vignetting and uneven lighting, an illumination profile was made from the mean image of each of the four image acquisition days and applied on the demosaiced images. Using the same color calibration cardboard as in Section 2.1.3, a color profile was manually calibrated for each day. RawTherapee version 5.8 was used to correct the white balance, brighten shadows, and enhance the appearance of the images. This included the use of exposure compensation, gamma correction, increased contrast, and chromaticity in CIE L*a*b* space, brightening of shadows using built-in filters, sharpening, edge enhancements, and micro-contrast.

### 2.3. Synthetic Image Dataset with Hierarchical Labels

Hierarchically labeled synthetic images provide a multitude of benefits compared to a flat real dataset of similar manual labor. Namely: (1) 100% pixel accurate labels, (2) compute-limited sample size as opposed to labor-limited, (3) controlled data distributions and biases, (4) emphasis on under-sampled parts (e.g., clover flowers) using intra-class labels.

For training the convolutional neural networks for semantic segmentation, the publicly available GrassClover dataset was used [16]. This dataset consists of 8000 photo-realistic synthetic images of grass clover mixtures with hierarchical species and instance labels. The synthetic images was made from a pool of 230 digitally cut-out plant samples (149 clover samples, 55 rye grass samples, and 26 weeds.), used to populate soil images, until a desired leaf area index was reached. The species sampling distribution was varied per image to mimic real world variations in grass clover fields. A synthetic image sample from the GrassClover dataset is shown in Section 3.3.2.

### 2.4. Image Annotation

The existing data-set of 15 labeled image crops by Skovsen et al. [16] is used for evaluating the per pixel image recognition performance. In this data-set, 15 image crops of 1000 × 1000 pixel were labeled per pixel into five classes: Soil, white clover, red clover, grass, and weeds. Ten of those images originate from densely grown plot trial sites A and B with biomass clover contents evenly distributed between zero and 100 percent. The remaining five labeled images originate from sparsely vegetated grass clover fields in October captured with the ATV platform.

## 3. Methods

### 3.1. Data-Driven Canopy Image Segmentation

Consistent with previous research in the field, the biomass composition of the mixed crop is estimated based on the visible canopy in a top-down camera view [2,3,7,8,10,19]. Setup as a semantic segmentation task, where every pixel is discretely classified, every biomass image is described by $9\times 10^{6}$ plant species classifications. The method of predicting the species composition in the biomass is then based on the detected area of each crop, corresponding to the number of pixels of the given image recognition class.

### 3.2. Neural Network Architecture

**Figure 4:**
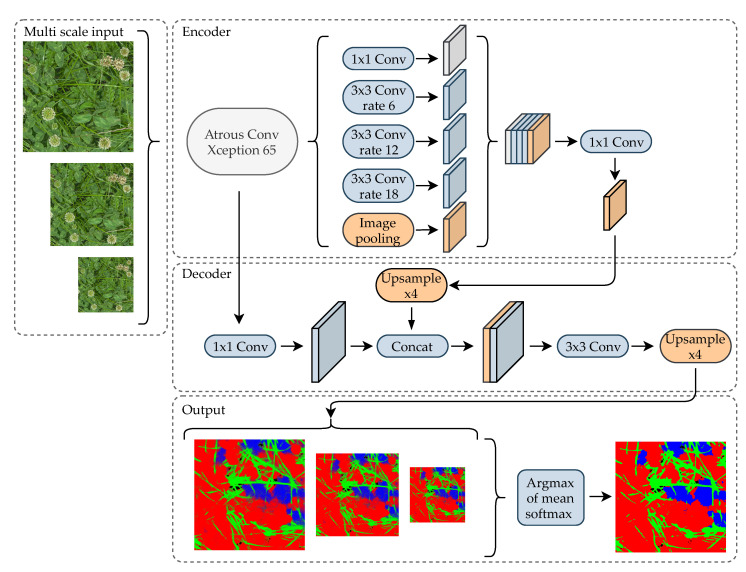
Overview of the Deeplab v3+ [20] neural network architecture. Features from the Xception 65 [21] based backbone is concatenated at multiple scales in the encoder using spatial-scale adjustable atrous filters. The decoder combines higher resolution features from within the Xception 65 with the multi-scale features to produce a spatially improved semantic segmentation. To further improve scale-invariance, images are processed at three image scales and averaged to a final class probability map. The encoder–decoder model architecture is illustrated with similarities to the original publication [20].

From demonstrated success in other domains, and the inherent use of multi-scale feature processing, the DeepLabv3+ [20] with a backbone network of Xception-65 [21] was selected. The key elements of the model architecture are illustrated in Figure 4. The encoder–decoder architecture is designed to refine semantic segmentations along object borders, by utilizing intermediate features at a spatial higher resolution. While this is commonly used, the feature processing at multiple scales using a spatial-scale adjustable atrous filter can potentially increase the robustness towards varying growth stages and plant sizes in the grass clover domain. To further improve scale-invariance, each image is processed at three scales, namely 100%, 75%, and 50%, and combined to a single semantic segmentation result.

### 3.3. Training Procedure

The convolutional neural network (CNN) architecture was trained in Tensorflow version 1.15.0 based on the official deeplab model repository [22]. Changes were made to (1) translate hierarchical labels into prospect classes during preprocessing, (2) introduce intra-class loss weighting, and (3) extend the image augmentation pipeline.

Following the principle of Skovsen et al. [12], two separate model instances were trained on the same hierarchical data and combined in a two-stage classification process: Model 1 was trained to discriminate between soil, clover, grass, and weeds. Model 2 was trained to discriminate between red clover and white clover without regards to image parts outside of the two classes. Model 2 can thus be used for further classification of the areas that model 1 has classified as clover, so that the clover species can be determined. In addition to the easier control of class biases, it also allowed the use of clovers without species annotations in the training process. This increased the training material, which contributed to the creation of a robust grass-clover segmentation model.

Although this structure doubles the total inference time, it permits a direct comparison with the existing literature in the field when ignoring the second stage.

The models were initialized with weights from a pre-trained Xception65 trained on ImageNet [23], followed by a deeplab3+ pretrainining on MS COCO [24].

Both models were trained with the same set of hyper parameters: Adam optimizer, learning rate of $1\times 10^{-5}$, weight decay of $4\times 10^{-6}$, 20,000 iterations, atrous rates of 6, 12, and 18, decoder output stride of 4 pixels, crop size of 768 × 768 pixels, and a batch size of 44, evenly distributed on clones on each of four Nvidia Quadro RTX 8000 GPUs.

The aim of this work was to create an image recognition model that handles images from ordinary grown fields. However, training entirely on synthetic data introduces the risk of overfitting on a domain, different from the target domain. To reduce the decoupling between the artificial and the real world, three strategies were employed, namely sub-class weighting, aggressive image augmentation, and style-transfer augmentation. While sub-class weighting and image augmentation were employed to improve the performance in generic real world conditions, style transfer was introduced as a semi- or unsupervised approach to deal with unfamiliar conditions, not present in the training dataset. In principle, this could reduce the need to manually extend the training data when new sampling conditions arise.

#### 3.3.1. Sub-Class Weights

Based on the hierarchical data distributions of the training data [16], a default class weighting scheme was applied to reduce biases towards over-represented (sub)classes and give emphasis to rare cases.

(1) $$w_{c}=w_{ce}\log\left(\frac{N_{c}}{N_{sum}}\right)$$

where $w_{c}$ is the weight of class *c*, $N_{c}$ is the total number of pixels in class *c*, $N_{sum}$ is the total number of pixels in the dataset, and $w_{ce}$ is a hyper parameter to control emphasis of the cross entropy loss.

In the case of red clover leaves and white clover leaves, which combined makes up half of the image data, an extension was made to regularize the weights based on the overall presence of clovers in the dataset. Weeds species were trained with a common weight, based on the combined weed coverage.

The final class weights used for the training of the CNNs were 3.97, 1.46, 2.86, 6.07, 1.46, 6.68, 6.07, 6.07, 6.07, 8.58, 2.41, 13.28, 0.88, and 1.46 corresponding to soil, clover, grass, weeds, white clover, red clover, dandelion, shepherds purse, thistle, white clover flower, white clover leaf, red clover flower, red clover leaf, and unknown clover leaf, respectively.

#### 3.3.2. Aggressive Image Augmentation

**Figure 5:**
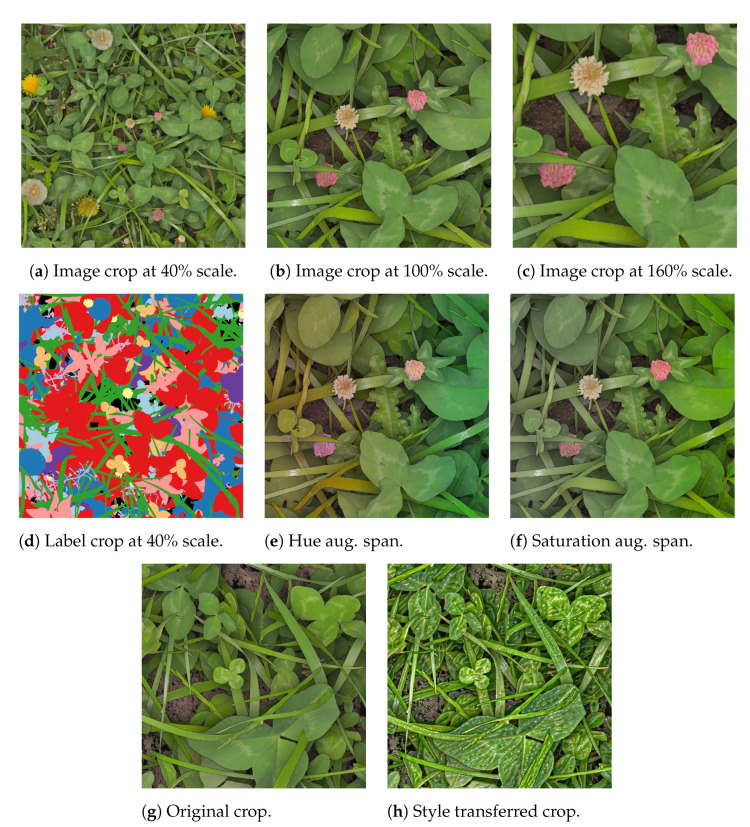
Illustration of image augmentations applied on the synthetic image crops during training of the model. (**a**–**f**) represent online augmentations throughout the training process; (**g**,**h**) represent style transfer of dew artifacts, used for offline-augmentation of synthetic images in subsequent finetuning of the 1st stage model.

In order to make the GrassClover dataset consistent with the real images, the resolution was initially set to fit a ground-sampling distance of 6 pixels per mm. However, to increase the robustness of the semantic segmentation outside of the evaluated scope, an aggressive augmentation strategy was introduced. The image scale of the training images was uniformly scaled by 60% as illustrated in Figure 5a–c. This was designed to (1) recognize grasses and clovers across growth stages, and (2) to simulate different camera sensors sizes, focal lengths, and camera to canopy distances. To reduce the reliance on welldeveloped images from manual calibrations, random augmentations of brightness, contrast, hue and saturation were also applied. The augmentation span of hue and saturation is visualized gradually in Figure 5e,f from left to right.

#### 3.3.3. Style Transfer Augmentation to Create Weather Condition Invariance

**Figure 6:**
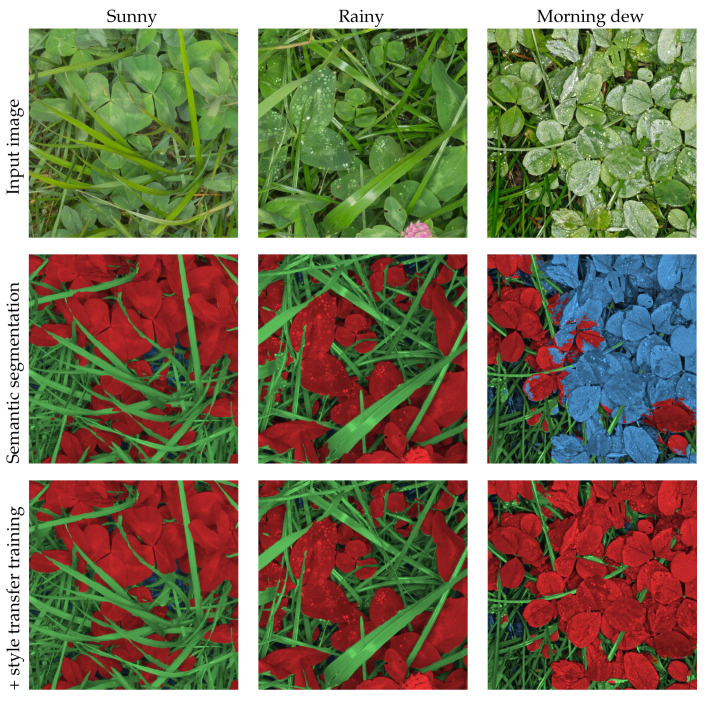
First row: Illustrative 900 × 900 pixel image crops of the weather conditions effect on image quality depending on droplet size. (**left**) Sunny condition without droplets. (**center**) Although the large droplets from rain are visible, the captured leaf texture is comparable to sunny conditions. (**right**) In dewy conditions, especially the clover leaf texture appearance is highly affected by the specular reflections from the camera flash. Second row: The semantic segmentation results with a model trained with traditional image augmentation. Third row: The semantic segmentation results when including style transferred dewy conditions into the synthetic training data. Green is grass, red is clover, and blue is soil and background.

Changing weather conditions during the acquisition of the 915 biomass sample pairs introduced diverse visual effects. This means that some plants have strongly reflective leaves due to rain or morning dew, as illustrated in Figure 6, where sunny, rainy, and dew conditions are compared. To increase the robustness of the trained model, and improve invariance towards moist and reflective leaves, style transfer was used to transfer the texture of moist leaves to training images containing dry leaves.

The publicly available implementation of GLStyleNet [25] was used to perform the style transfer. The hyper parameters were initially tuned to transfer weather-induced artifacts from real to synthetic images, while ensuring consistency across plant species and positions in the synthetic image. Since class label images were not augmented, consistency in the content before and after the style transfer was important to not introduce label errors in the training data.

Thirty-two images with severe reflections from morning dew were selected to provide image styles. Using GLStyleNet, 493 synthetic image crops of 1600 × 1600 pixels were then augmented to express wet weather conditions in an unsupervised approach.

Model 1, which handles classification of soil, clover, grass, and weeds, was subsequently fine-tuned for 6000 iterations at a learning rate of $5\times 10^{-6}$ on a combined dataset of the GrassClover synthetic images and style transferred synthetic images. Here, the style transferred images represented 6% of the training data.

### 3.4. Validation in Large Scale Mapping

The 29,848 images, distributed across 16 fields, as shown in Table 2, were processed with the 1st stage model in full resolution of 2054 × 2456 pixels to segment the images into grass, clover, weeds and soil. Due to omission of red clover in the fields, the 2nd stage model was not included for large scale mapping. Using QGIS v.3.2.3, the relative clover content of each image position was interpolated to a 5 × 5 m grid within the field boundary of each mapped field. Given the large number of samples, at approximately 150 samples per hectare, ordinary kriging interpolation, inverse distance interpolation, and linear interpolation resulted in comparable species maps. However, due to the relationship between soil conditions and species competition, and the long tradition of kriging interpolations for soil characteristics mapping, ordinary kriging was used.

Evaluation of the large scale biomass composition mapping in crop mixtures introduces new challenges for the research community. With mapped areas five orders of magnitude larger than the manually fractioned biomass samples of 0.25 m^2^, the biomass composition at field scale cannot be performed manually through fractioning into plant species. Sparsely distributed biomass samples, providing the exact biomass composition in sub-m^2^, might not be representative to a larger region due to local variations at multiple scales in the mixed crop.

No images from the ATV-based image acquisition platform have corresponding biomass samples. As a consequence, the predictive performance from canopy image to biomass composition, specifically for that camera system, cannot be evaluated directly. In order to validate a consistent segmentation accuracy of Model 1 on the ATV-based images, three other approaches were used: (1) Test on five pixelwise labeled image crops of 1000 × 1000 pixels of varied clover content. These images are included as part of the GrassClover semantic segmentation test set. (2) Qualitative semantic segmentations of the five image crops along with ground truth labels and previous work. (3) Qualitative results of semantic segmentation for each of the four days of data acquisition with a variation in both biomass quantity and composition.

## 4. Results

The result section is ordered into sections regarding semantic segmentation of images, biomass composition prediction using images, and large scale mapping using images.

### 4.1. Semantic Segmentation

The two cascaded models were evaluated for pixelwise classification on the Grass-Clover dataset [16] evaluation server with results stated in Table 3. The 1st stage model is segmenting the images into grasses, clovers, weeds, and soil, followed by the 2nd stage model, classifying the clovers into species. Making use of the separation between the 1 and 2nd stage, a cascaded model can consist of any 1st and 2nd stage models, independent of CNN architecture or training procedure. In this way, a 2nd stage DeepLabv3+ model can be substituted by a previous FCN-8s 2nd stage model to isolate the contributions of each work.

The baseline FCN-8s based model [12] reached a mean Intersection over Union (IoU) of 55.0%. The DeepLab3+ST cascade demonstrated a notably improvement of the mean IoU of 13.8 percentage points, primarily driven by high improvements in both grass, weeds, and soil segmentations. Employing a DeepLabv3+ST → FCN-8s cascade further improved the mean IoU by 2.6 percentage points to a mean IoU of 68.4%. Since all edge-sensitive segmentations are performed in the first stage, with the exception of overlapping clover species, the FCN-8s model appears to generalize better to the task of clover species discrimination, but at a general cost of spatial accuracy in semantic segmentation.

**Figure 7:**
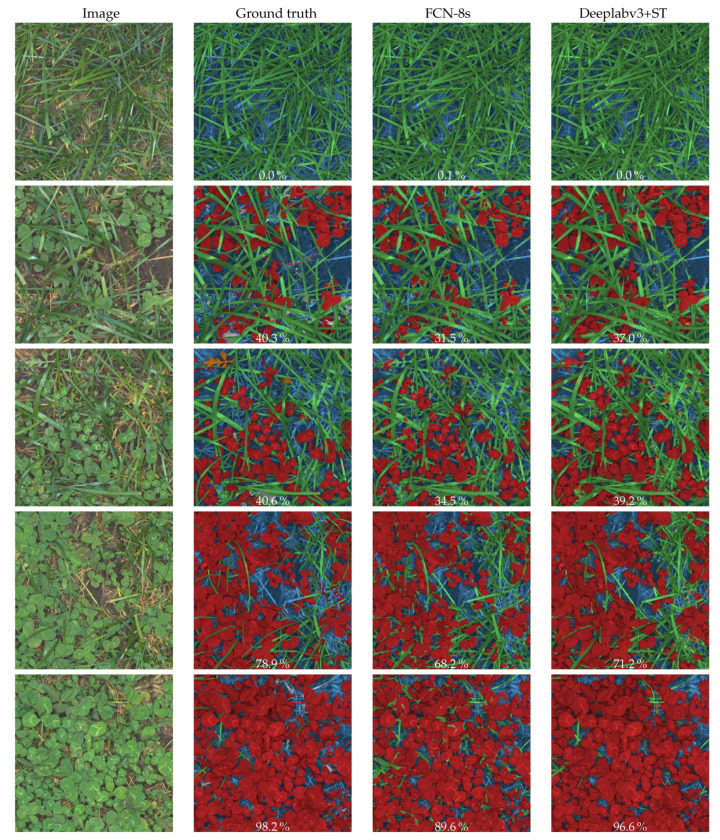
Comparison between ground truth labeled images crops of 1000 × 1000 pixels and corresponding semantic segmentations from FCN-8s and Deeplabv3+ models. The images originate from the fall acquisition using the large scale image acquisition platform and vary in clover content. Each row represents one image. The derived clover coverage, relative to the detected canopy, is written in white text. Red is clover, green is grass, blue is soil+background, orange is weeds, and grey is unknown. The predictions from the Deeplabv3+ based model are consistently closer to the ground truth than the FCN-8s based model.

Figure 7 compares the FCN-8s model from [15] and the DeepLabv3+ST models on five images with corresponding ground truth annotations from the large scale image acquisition platform. Since clover species discrimination on the mapped fields is peripheral due to a single seeded clover species, only the first stage is compared. Although the FCN-8s model detected most clovers and grasses, the rugged overextended grass segmentations and missed clover leaves led to a general underestimation of the clover content. The predicted clover coverage of the canopy in each image, noted in white, was consistently better predicted with the DeepLabv3+ST model. The improved scale-invariance using the DeepLabv3+ST model is believed to be an important step to handle even the smallest clovers in the images. The clearest discrepancies between the ground truth and the DeepLabv3+ST model are (1) the misclassified weed in the third row, (2) disagreement in the decision boundary between grass and background for dry grasses, most apparent in row 4, and (3) misclassifications of clover stems as grasses.

**Figure 8:**
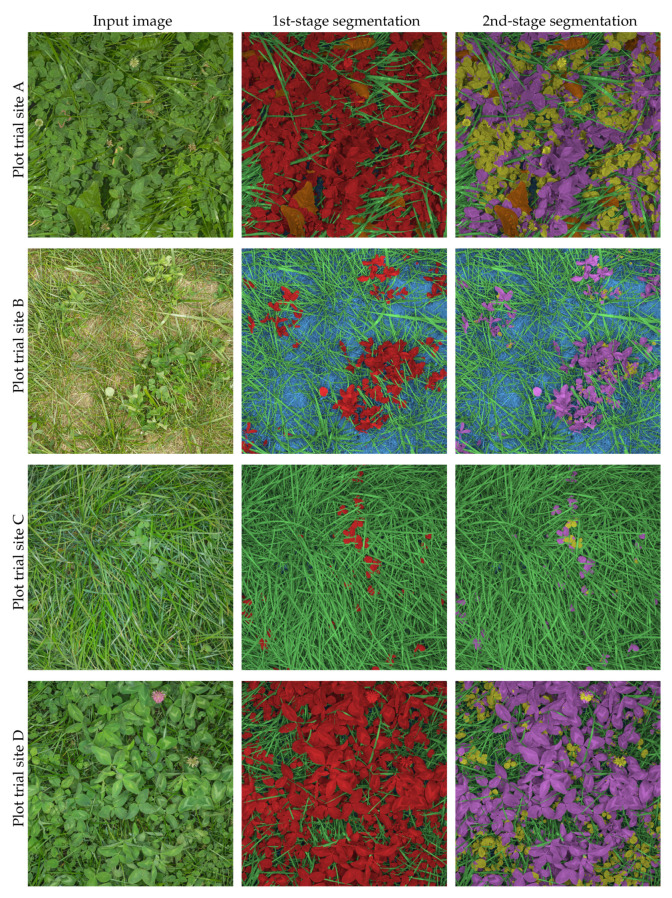
Qualitative samples of semantic segmentation on four plant samples of varied yield and biomass compositions using DeepLabv3+ST followed by FCN-8s for clover species discrimination. First column: Input red green blue (RGB) image of 3000 × 3000 pixels. Second column: 1st-stage pixelwise classification of image into soil (blue), clover (red), grass (green) and weeds (orange). Third column: 2nd-stage pixelwise classification of image into soil (blue), red clover (purple), white clover (yellow), grass (green), and weeds (orange).

Figure 8 demonstrates qualitative examples of the DeepLabv3+ST → FCN-8s cascaded semantic segmentation on biomass samples from each of the four plot trial sites. Without directly comparable ground truth images, errors in the 1st stage segmentation are difficult to spot. Even the heavily occluded leaves in the third row are being correctly detected and segmented. The errors in the output of the 2nd stage are more obvious. As described in Table 1, plot trial site C was established without red clovers—here, the majority of the clovers were falsely classified as red clover. In the remaining images, the clover species appeared to be better separated, with a couple of clovers being misclassified in each plot.

### 4.2. Biomass Composition Prediction

Biomass composition predictions was made for all 915 biomass samples from the cascaded model predictions, similar to those shown in Figure 8. In addition to evaluating the end-goal of predicting the biomass species composition in grass clover mixtures, it allowed the semantic segmentation models to be tested indirectly on a much more diverse dataset which included four locations, three seasons, three camera setups, multiple seed mixtures, and different weather conditions.

Table 4 compares the coefficient of determination (R^2^) between the predicted canopy segmentations and the corresponding biomass fractions using a first order linear model on four different model cascade combinations. Focusing on the 1st stage image segmentation into clover, grass, and weeds, there was a clear improvement moving from the FCN-8s model to the DeepLabv3+ of 4.5 percentage points for clover fraction predictions. Since the grass fraction prediction was not improved, the improvement presumably came from better separation of clovers, weeds, and soil in the biomass images.

**Figure 9:**
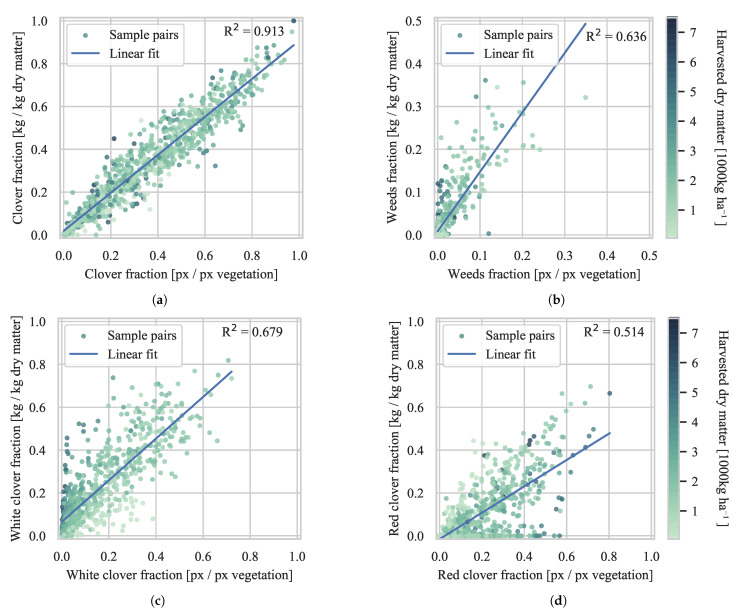
Visualization of the correlation between the predicted visual canopy fractions and the corresponding dry matter fractions. The top row represents 915 biomass samples. The second row represents a reduced set of 752 biomass samples, due to omitted clover species annotations in most biomass samples from plot trial site B. (**a**–**d**) represent clover, weeds, white clover, and red clover fractions, respectively.

The introduction of style-transfer augmentation of synthetic data to improve weather condition invariance, as described in Section 3.3.3, contributed to a major improvement on the results. An increased R^2^ of 2.7, 3.2, and 10.8 percentage points were observed for clover, grass, and weeds prediction, respectively. Similar to the semantic segmentation results of Table 3, the DeepLabv3+ did not improve the clover species prediction performance. Figure 9 shows four of the canopy segmentation to biomass fraction correlation plots of the DeepLabv3+ST → FCN-8s model cascade, from which the presented R^2^ originates. The linear correlations from canopy image to the *secondary* biomass fractions of weeds and specific clover species were not too reliable, in part due to the varying vertical distributions of the fractions in the sward. However, the correlation of the *primary* fractions of clovers and grasses demonstrated a convincing robustness across the four locations, three seasons, three camera setups, multiple seed mixtures, and changing weather conditions.

#### 4.2.1. Evaluation of Generalization

Computer vision techniques can often be *over*fitted to the evaluated conditions in a degree that penalizes generalizability. This is especially true in areas of expensive sample acquisition, such as biomass sampling and cumbersome fractioning of mixed crops. As a consequence, the evaluated samples are often limited in quantity or variation. To evaluate the generalizability of recent methods, the 915 biomass samples were organized according to (1) acquisition date, (2) experimental site, and (3) seasonal cut no. The correlations between the detected canopy clover fraction and the biomass clover fraction for each set of samples were then determined, as shown in Table 5. While a low correlation in a single acquisition can indicate difficult weather conditions (e.g., site A, cut 4), a drop in correlation when including all cuts or sites indicates a lack of generalization.

Morphological filtering from Mortensen et al. [9], similar to [5,6,7,8], provided a good predictor on individual acquisition dates, particularly in mixtures of ryegrass and white clover (site C, R^2^ ≈ 88%). When red clover was added in the mixture (site A, B, and D) the average correlation was significantly reduced (R^2^ ≈ 65%). The inherent sensitivity to scale in morphological filtering was likely insufficient to equally detect the different sized leaves of the two clover species. When used for multiple acquisition dates, the average correlation was reduced to R^2^ = 48.2%, R^2^ = 51.4% and R^2^ = 36.9%, across cuts, sites and all samples, respectively.

The deep learning based FCN-8s model from Skovsen et al. [16], similar to [3,12] demonstrated better use as a predictor on each of the 18 acquisition dates, individually. However, the biggest improvement, compared to morphological filtering, was the greatly improved generalizability. With a correlation across all 915 biomass samples of R^2^ = 84.1%, the data driven approach, albeit cumbersome to train, provided a single model, suitable for multiple locations and species compositions for the duration of the productive season.

The DeepLabV3+ST further improved the predictive performance from the analyzed canopy image. Compared to FCN-8s, the biggest improvements for individual sites were those of lower correlations (R^2^ < 80%), but all acquisitions demonstrated a higher correlation. Five out of the eighteen acquisition dates demonstrated an R^2^ < 90%, but four of those were sampled in rainy (site D, cut 2; site D, cut 5) or dewy (site A, cut 4; site B, cut 4; site D, cut 5) weather conditions. Across all 915 biomass samples, the DeepLabv3+ST demonstrated an R^2^ = 91.3%, and, within the common mixture of ryegrass and white clover (site C), the correlation was increased to R^2^ = 94.6%. The presence of red clover in the mixtures had a small impact on the correlation, but this was more likely the result of a harder task, than unbalanced detection of the two clover species.

#### 4.2.2. Comparison with Previous Studies

Table 6 compares the presented results in relation to previous studies in image based biomass clover fraction prediction. As published results are highly dependent on the evaluated dataset, this comparison includes a dataset description along with the method. To support fair comparisons across the datasets, important factors related to (1) occlusions (biomass range), (2) scale invariance (biomass range), (3) image quality (GSD), and (4) generalizability (no. cuts and evaluation sites) were included. While Table 6 highlights the strong dependency on a good dataset, it provides valuable insights into design choices such as image quality.

The morphological filtering implementations in [8,9], resulted in correlations of R^2^ = 85% and R^2^ = 54.8%, respectively, on two different datasets. While the difference is high, the extended biomass range and number of seasonal cuts in the second dataset demonstrated the main shortcomings of the method. Himstedt et al. [8] referred to this shortcoming as a need for self-adjusting parameters for the method to be applicable under variable field conditions.

The FCN-8s implementations in [2,16], presented correlations of R^2^ = 79.3% and R^2^ = 92.8%, respectively, on two different datasets. While the latter dataset was more extensive in terms of biomass range and number of seasonal cuts, the perceived image quality was also higher. The most natural explanation for the difference in correlation is the higher image resolution and quality, which would allow for improved species recognition in shaded parts of the canopy and along leaf edges. However, due to the data driven approach of deep learning model optimization, part of the difference might originate from suboptimal training data or hyper parameters during model training.

The LC-Net implementation in [2] improved the R^2^ correlation by 3.2 percentage points, relative to the FCN-8s architecture on the same dataset. This improvement was contributed to (1) better spatial accuracy of image segmentations using an encoder–decoder architecture, and (2) incorporating local context features in form of a local context pyramid pooling (LCPP) block [2].

The DeepLabv3+ST model implementation in this work surpassed all previously published results in ryegrass and white clover mixtures with an R^2^ = 94.6% on the extensive dataset of 240 biomass samples across five seasonal cuts. Although the DeepLabv3+ model architecture was not directly compared with the LC-Net model, the combination of dataset and model improved the correlation by 12.1 percentage points.

#### 4.2.3. Test Set Validation

**Figure 10:**
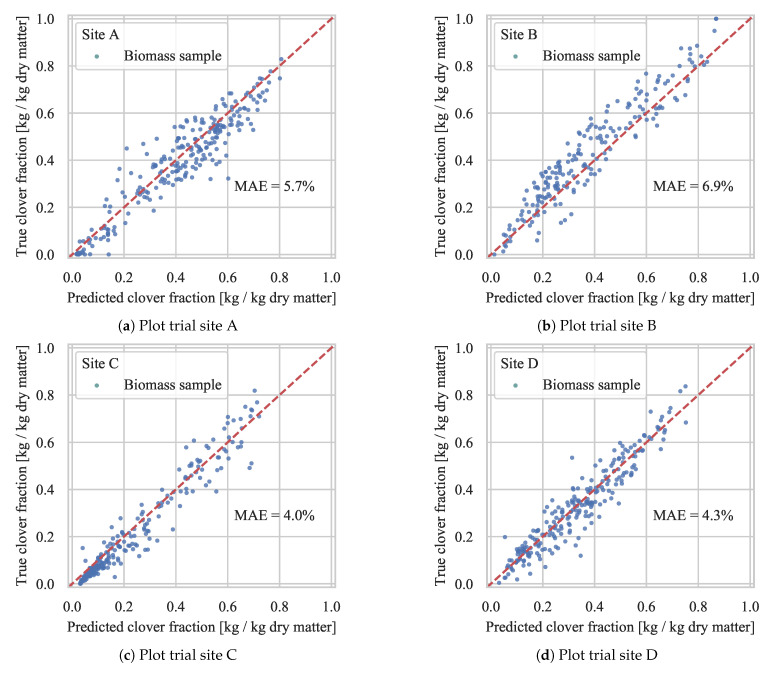
Test of biomass clover fraction prediction at each plot trial site, based on a first order linear model fitted to the remaining three sites. The site-specific mean absolute error, denoted MAE, is printed in each subfigure.

To test the predictive clover content accuracy of the proposed DeepLabv3+ST model on unrelated test locations, the biomass samples were split into four folds, corresponding to the four plot trial sites. Based on a first order linear model, fitted to the data from three sites, the clover fraction for each biomass sample at the fourth site was predicted. The results for each plot trial site are shown in Figure 10. With a mean absolute prediction error ranging from 4 to 6.9 percentage points, the prediction accuracy was not heavily degraded from testing at a separate location. At plot trial sites A, C, and D, the biomass sample predictions were evenly distributed above and below the true values, suggesting a good fit, and that an increase in acquired samples (e.g., on a densely sampled field) would lower the average prediction error. Although each plot trial site differed in the seeded species composition, this had no visible effect on the derived clover content prediction in the tests.

### 4.3. Biomass Yield Prediction

**Figure 11:**
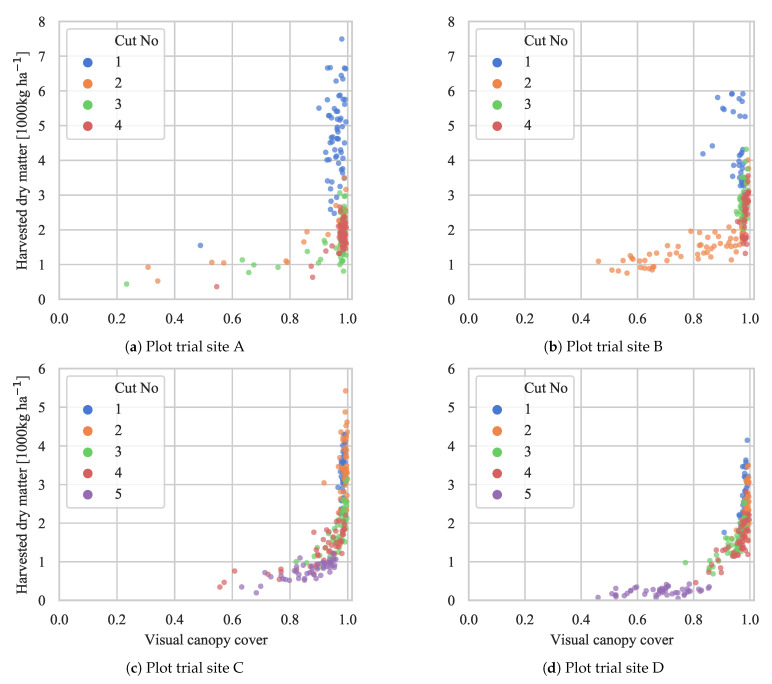
Comparison between the predicted visual canopy coverage and the corresponding yield in the biomass samples. The data are visualized for each plot trial site individually to emphasize local trends. (**a**–**d**) correspond to plot trial sites A, B, C, and D, respectively.

Comparable to the biomass fraction estimates of clover, weeds, red clover, and white clover in Figure 9, the biomass yield was plotted relative to the detected canopy cover in Figure 11. Similar to the multispectral normalized difference vegetation index (NDVI), the coverage saturated at biomass yields above 2000 kg ha^−1^. Locally, at each plot trial site, the detected canopy coverage explained a gradual increase in yield, with the most clear trends at sites C and D. The difference in trends between the sites is thought to originate from a combination of species composition, growing conditions (i.e., water, soil and weather), establishing method, and cutting height. By leaving the interpretation up to the agricultural advisor or crop consultant, we demonstrate field maps of canopy coverage in Section 4.4.

### 4.4. Large Scale Mixed Crop Mapping

**Figure 12:**
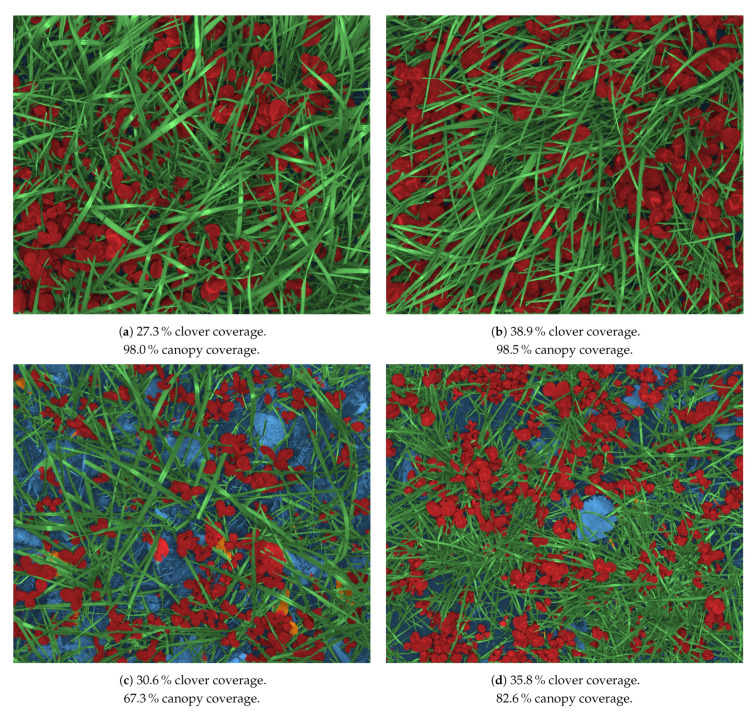
Pixelwise classification of the four large scale image acquisition samples from Figure 3. Blue is soil+background, red is clover, green is grass, and orange is weeds. The caption for each subfigure exemplifies the two automatically predicted metrics used for large scale mapping.

Using the 1st stage DeepLabv3+ST model, each large scale acquired image was semantically segmented as demonstrated in Figure 12. From each segmented image, the clover coverage, relative to the canopy coverage, was extracted, along with the canopy coverage relative to the image size. The two metrics are exemplified for each subfigure of Figure 12. Based on the 29,848 canopy images collected over 16 fields of a combined 225 ha (Table 2), two sets of maps were made, corresponding to each of the two extracted metrics.

**Figure 13:**
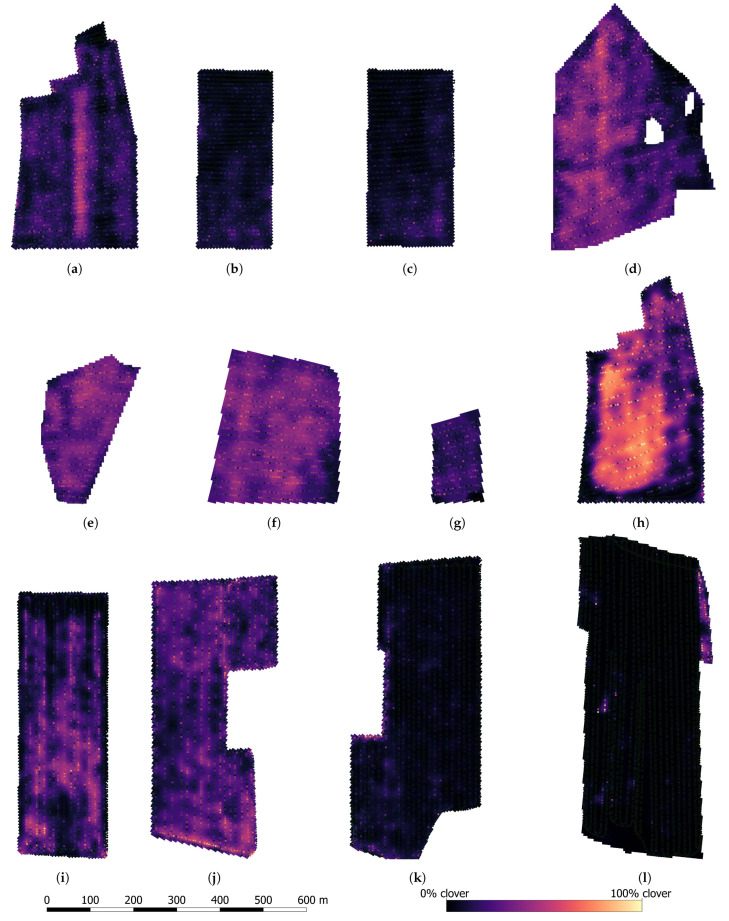
Visualization of the visual clover fraction in a subset of the large scale mapped fields. Fields (**a**–**g**) were sampled in May. Fields (**h**–**l**) were sampled in October. Each square unit represents a 5 × 5 m interpolated clover fraction. Each dot represents the visually predicted clover fraction in a corresponding image.

Twelve of the clover content mapped fields are shown in Figure 13. The maps are interpolated, but the individual sample predictions are overlayed for comparison. In May, corresponding to Figure 13a–g, three fields (d–f) demonstrated a high amount of clover, suggesting a reduced need for applied nitrogen fertilizer. Two fields (b,c) were consistently low in clover. The first field (a) contained a stripe of increased clover content—the result of a no-nitrogen-application experiment in the preceding spring. In October, four (i–l) of the five fields were recently established, and were mapped preceding their first yielding season to determine the need for nitrogen application from the start. Two fields (k,l) demonstrated insignificant amounts of clover. This was possibly due to clover establishment failure, pointing to a need for either supplementary clover seeding, or to maximize the nitrogen fertilization to maintain a high-yielding field. One field was mapped in both periods (a,h), five months apart. In this period, the clover content in the center of the field increased dramatically, while diminishing close to the field border.

**Figure 14:**
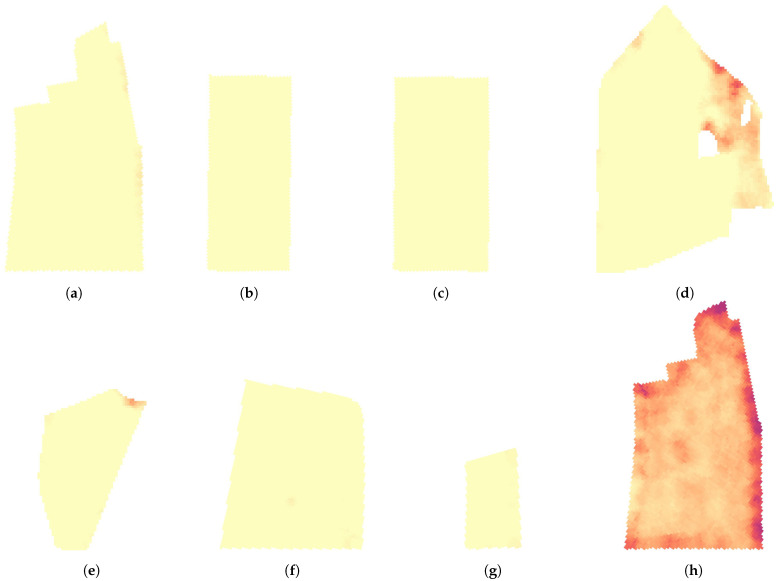

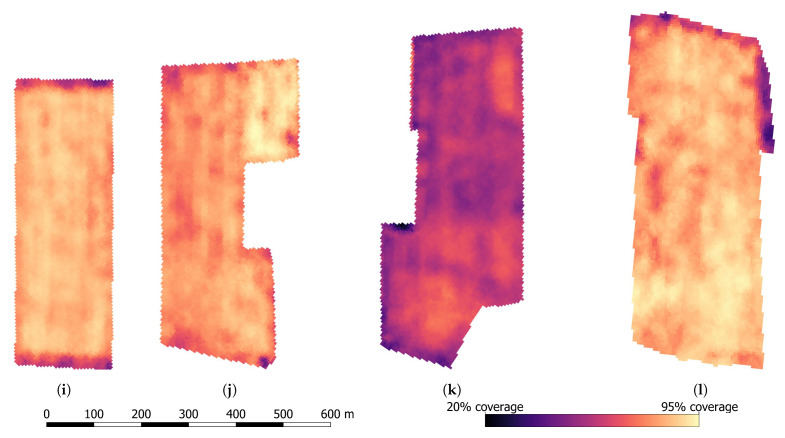
Visualization of the predicted canopy coverage in a subset of the large scale mapped fields. Fields (**a**–**g**) were sampled in May. Fields (**h**–**l**) were sampled in October. Each square unit represents a 5 × 5 m interpolated value.

The same twelve fields were similarly mapped for canopy coverage in Figure 14. In May, only one field (d) did not demonstrate a consistent canopy coverage, explained by a patch of wetland (cropped out of the map) with muddy surroundings. In October, the coverage maps were not saturated, and demonstrated insights in the locally established mixed crop. Reduced crop growth was visible in the headland of (i). Field (k) stood out with a clearly lower canopy coverage, repeating the suggestion of reseeding the field. Some artifacts from sampling along the field tracks are visible in two fields (i,j), in the form of broad vertical stripes. This is caused by periodic sampling in local variations caused by tractors or implements. Most other fields were sampled either at an angle, or perpendicular to the field tracks to reduce this problem.

## 5. Discussion

With the popularity of deep learning and data driven methods, improvements in state-of-the-art model architectures for general computer vision can often be transferred to agronomy. However, as the complexity in the experimental design increases, so does the difficulty in obtaining, reproducing, and comparing results. In the chain of events from biomass sampling, to image acquisition and computer vision, inaccuracies at a single point reduce the observed result in the end. With the introduction of data driven models, additional potential sources of error have been introduced in the form of insufficient training data, inaccurate image labels, and suboptimal model hyper parameters. While deep learning can provide great improvements for computer vision in agronomy, as demonstrated in this work, care must be taken to: (1) acquire a representative dataset, adequately spanning the problem at hand, and (2) recognize the impact of camera systems and optimal lighting for computer vision tasks. Although we have presented state-of-the-art results in image based prediction of the biomass clover fraction; this is also the result of a high quality dataset not being the limiting factor in the analysis. With the recent release of the GrassClover dataset [16], we hope to see it being used as a common reference for fair comparisons of future computer vision methods on mixed crops.

The proposed transition from a VGG16 [13] based FCN-8s [11] model to an Xception-65 [21] based DeepLabv3+ [20] model for grass clover segmentation greatly improved the biomass predictive accuracy. However, the reduced model generalization for detecting clover species suggested that the improved model was increasingly sensitive to conditions outside of the grass clover training data. This might explain the reported lack of scaleability in [2], the observed gains from using style transfer to expand the variability in the training data, and the inferior accuracy in discriminating clover species. Due to the nature of transfer learning, the two model backbone architectures cannot be compared directly. However, as the VGG16 and the Xception-65 model backbones contain approximately 138M and 41M parameters, respectively, it is possible that pretraining on ImageNet leads to more specialized feature extractors in the smaller, newer model. While agronomy is only vaguely represented in ImageNet, more specialized features for ImageNet are not necessarily a benefit when dealing with small datasets in agronomy. The DeepLabv3+ was additionally pre-trained on MS COCO for semantic segmentation, to pre-train the extensive model parameters surrounding the Xception-65 backbone in the encoder–decoder architecture. While this additional pretraining might have deteriorated the backbone parameters, it is likely that the rest of the DeepLabV3+ model was limited in generalization caused by the relatively smaller complexity of image classes in MS COCO.

With the increased reliability of accurate clover content predictions from images, camera based prediction systems are in rising demand. In contrast to manually fractioned biomass samples or inconsistent visual inspections, computer vision opens up for large scale research experiments in the form of (1) hundreds or thousands of plots, or (2) plot sizes large enough to be used for satellite imagery. Albeit the prediction error is not zero in the proposed method, an increased number of repetitions will not only reduce the average image prediction error, but also reduce the consequence of random events occurring in the physical research plots.

The detailed evaluation of model generalization in Table 5 cemented one of the major improvements that deep learning has contributed to this field. The demonstration of a single model to be used in any of four different species mixtures, at biomass levels ranging from 50 to 7500 kg ha^−1^ and without day to day adjustments, was not possible nine years ago. Although future investigations are still needed to evaluate the need for high quality image data, this work has demonstrated the feasibility of automated species distribution mapping of mixed crops.

## 6. Conclusions

It has been shown that the Xception-65 based DeepLabv3+ model architecture can significantly outperform the earlier VGG16 based FCN-8s model for semantic segmentation of grass clover images into grass, clover, weeds, and soil. With an extended data augmentation scheme, including style transfer for environmental effects, the DeepLabv3+ model increased the semantic segmentation Intersection over Union (IoU) by 13.4 percentage points. The increased image segmentation IoU greatly improved the R^2^ of predicting the relative biomass composition from the color images. Evaluated on 915 canopy images with corresponding biomass samples, collected at four different experimental sites across three seasons, the grass and clover fractions were predicted with an R^2^ of 90.5% and 91.3%, respectively, between the two domains. In mixtures of only ryegrass and white clover, the correlation for clover fraction prediction was increased to a new state of the art of R^2^ = 94.6%.

Based on the evaluation of 29,848 spatially distributed high quality images across 225 ha, the local clover fraction of 16 grass clover fields was mapped, as well as the projected leaf area index. With the demonstrated large scale capabilities of highly accurate biomass composition predictions in mixed crops, it has been shown that computer vision sensors can provide reliable, valuable metrics into both research and management of mixed crops in real world conditions, without the need for ongoing hyper-parameter adjustments.

## Figures and Tables

**Table 1:** Comparison of the four plot trial sites. Plots in site A, C, and D were all established with a location-specific seed mixture, followed by nitrogen application trials to induce a varied clover content across the plots. Plots in site B were established with a wide range of commercially available seed mixtures, leading to a high variation between the plots, but inconsistent representation of the four species in the plots.

Plot Trial Site	A	B	C	D
Seeded plant species
Lolium perenne	✓	(✓)	✓	✓
× Festulolium		(✓)		✓
Trifolium repens	✓	(✓)	✓	✓
Trifolium pratense	✓	(✓)		✓
Herbicides		✓		
Soil type	Loamy sand	Sandy loam	Loamy sand	Coarse sand
Cuts per season	4	4	5	5
No. of plots at site	60	>200	48	48
Years since plot establishment	1–4	1–2	2	2
Sample years	2017	2017–18	2019	2019
Acquisition weather conditions
Sunny	✓	✓	✓	✓
Rain			✓	✓
Morning dew	✓	✓	✓	✓
Location
Latitude	56.4957	55.3397	55.5370	56.1702
Longitude	9.5693	12.3808	8.4952	8.7816
Camera system samples
Nikon D810A **+** 2× LED flash	179	83		
Sony a7 **+** ring flash	60	113	180	240
Sony a7 **+** 2× speedlight flash			60	
Total number of biomass samples	239	196	240	240

**Table 2:** Summary of the large scale image acquisition per field.

Farm	Field	Area [ha]	Acquisition Time [mm:ss]	Images	Density [Images ha^−1^]	Speed [ha hour ^−1^]
May 2018						
A	1	11.3	44:35	2223	197	15.3
A	2	18.6	67:01	3398	183	16.7
A	3	8.1	23:04	1188	145	21.1
A	4	7.1	26:50	1330	185	15.9
B	1	14.2	49:58	2025	143	17.1
B	2	4.8	17:50	723	148	16.1
B	3	9.2	40:43	1202	135	13.6
B	4	2.2	10:50	1202	135	12.2
Oct 2018						
A	1	11.3	34:38	1380	122	19.6
A	2	45.8	28:16	1422	31	97.2
A	3	16.9	49:25	2423	143	20.5
A	4	14.6	46:49	2170	148	18.7
A	5	12.5	44:11	2163	173	17.0
C	1	9.4	47:06	1878	200	12.0
C	2	20.5	78:12	3324	162	15.7
C	3	18.8	58:46	2999	160	19.2

**Table 3:** Mean and per class Intersection over Union for semantic segmentation on the GrassClover test set on the evaluation server. The baseline result is provided by the two hierarchically trained FCN-8s models [12]. ST represents the additional fine-tuning on style-transfer augmented image samples. The best result in each category is marked in bold type.

Cascaded CNN Models		Intersection over Union [%]					
1st Stage Model	2nd Stage Model	Mean	Grass	White Clover	Red Clover	Weeds	Soil
FCN-8s [16]	FCN-8s [16]	55.0	64.6	59.5	72.6	39.1	39.0
DeepLabv3 + ST	DeepLabv3+	65.8	**78.5**	62.3	75.0	**51.4**	**61.6**
DeepLabv3 + ST	FCN-8s [16]	**68.4**	**78.5**	**70.5**	**80.1**	**51.4**	**61.6**

**Table 4:** Comparison with Skovsen et al. [16] on the same dataset using coefficient of determination between analyzed canopy cover and relative biomass content for each class of species. Contrary to Table 3, this evaluation includes all 915 biomass samples in the comparison to maximize the extent of experimental sites, camera systems, seeded compositions, weather conditions, and seasons. The best result in each category is marked in bold type.

Cascaded CNN Models		Relative Biomass R^2^ [%]				
1st Stage Model	2nd Stage Model	Total Clover	Grass	White Clover	Red Clover	Weeds
FCN-8s [16]	FCN-8s [16]	84.1	87.2	61.1	**53.5**	46.1
DeepLabv3+	DeepLabv3+	88.6	87.3	64.8	44.9	53.8
DeepLabv3 + ST	DeepLabv3+	**91.3**	**90.5**	64.4	45.8	**64.6**
DeepLabv3 + ST	FCN-8s [16]	**91.3**	**90.5**	**67.9**	51.4	**64.6**

**Table 5:** Detailed comparison of the DeepLabv3+ST model with the FCN-8s model from Skovsen et al. [16] and the extended morphological filtering from Mortensen et al. [9] on the 915 biomass samples. The relative clover content prediction is evaluated using coefficient of determination between the detected clover fraction of canopy cover and relative clover content in the biomass, decomposed into individual experimental sites and seasonal cuts. The generalizability of each method can be observed by the drop in predictive performance moving from individual acquisition dates to aggregations across seasonal cuts and experimental sites.

		Relative Clover Biomass R^2^ [%]					
		Cut 1	Cut 2	Cut 3	Cut 4	Cut 5	All Cuts
Morph. filt.	Site A	71.8	81.3	79.9	36.3	-	19.1
	Site B	65.6	68.1	69.9	22.5	-	64.8
	Site C	92.7	89.2	75.5	91.5	88.9	54.8
	Site D	67.9	65.3	61.5	81.8	68.0	54.2
	All sites	36.4	26.4	59.3	58.3	76.4	36.9
FCN-8s	Site A	74.1	87.8	87.8	56.9	-	74.4
	Site B	90.7	84.3	87.3	79.6	-	84.9
	Site C	95.0	91.2	93.4	95.3	94.8	92.8
	Site D	90.9	84.8	92.6	91.2	68.7	86.1
	All sites	88.4	79.9	89.9	86.1	79.0	84.1
DeepLabv3+	Site A	82.1	94.4	95.1	67.0	-	87.8
	Site B	92.5	92.6	90.6	87.6	-	90.2
	Site C	95.5	93.4	95.4	97.5	95.6	94.6
	Site D	92.0	87.2	94.1	91.6	70.6	89.8
	All sites	91.2	90.7	92.8	91.4	85.3	91.3

**Table 6:** Comparison with previously published results on image based prediction of biomass clover fractions. For fair comparison, only mixtures with similar species are compared. Due to the scarcity of published results on ryegrass, white clover, and red clover mixtures, only mixtures of ryegrass and white clover (site C) were included in this comparison. When a data source is referenced, the corresponding R^2^ is reprinted from the data source. wc and rg is white clover and ryegrass, respectively. BM is biomass. GSD is ground sampling distance.

Method	Data Source	BM Range [1000 kg ha^−1^]	GSD [mm^−1^]	No. Samples	No. Cuts	Eval. Sites	Species Mixture	Clover R^2^ [%]
Morph. filtering [8]	[8]	< 2.8	2	24	3	1	wc, rg	85
FCN-8s [2]	[2]	1.0–3.3	2–3	70	2	1	wc, rg	79.3
LC-Net [2]	[2]	1.0–3.3	2–3	70	2	1	wc, rg	82.5
Morph. filtering [9]	Site C	0.2–5.4	6	240	5	1	wc, rg	54.8
FCN-8s [16]	Site C	0.2–5.4	6	240	5	1	wc, rg	92.8
DeeplabV3 + ST	Site C	0.2–5.4	6	240	5	1	wc, rg	94.6

## Data Availability

Publicly available datasets were analyzed in this study. This data [16] can be found here: https://vision.eng.au.dk/grass-clover-dataset. Additional data samples were collected and analyzed in this work. These samples will be made publicly available in an updated version of the same dataset.
